# Jun, an Oncological Foe or Friend?

**DOI:** 10.3390/ijms26020555

**Published:** 2025-01-10

**Authors:** Zuhayr Jafri, Yue Li, Jingwen Zhang, Connor H. O’Meara, Levon M. Khachigian

**Affiliations:** 1 Vascular Biology and Translational Research, Department of Pathology, School of Biomedical Sciences, Faculty of Medicine and Health, University of New South Wales, Sydney, NSW 2052, Australia; 2 Division of Head & Neck Oncology and Microvascular Reconstruction, Department of Otolaryngology, Head & Neck Surgery, University of Virginia Health Services, Charlottesville, VA 22903, USA; 3 Department of Otolaryngology, Head & Neck Surgery, Australian National University, Acton, ACT 0200, Australia

**Keywords:** Jun, AP1, transcription factor, cancer

## Abstract

Jun/JUN is a basic leucine zipper (bZIP) protein and a prototypic member of the activator protein-1 (AP-1) family of transcription factors that can act as homo- or heterodimers, interact with DNA elements and co-factors, and regulate gene transcription. Jun is expressed by both immune and inflammatory cells. Jun is traditionally seen as an oncoprotein that regulates processes involved in transformation and oncogenesis in human tumours. This article examines the traditional view that Jun plays a permissive role in cancer development and progression, whilst exploring emerging evidence supporting Jun’s potential to prevent immune cell exhaustion and promote anti-tumour efficacy.

## 1. Introduction

AP-1 represents a family of dimeric transcription factors that bind DNA through their bZIP domains and regulate transcription. AP-1 proteins (e.g., Jun/Fos, Jun/BATF) can form ternary complexes with other types of transcription factors such as nuclear factor of activated T cells (NFAT) and interferon response factor (IRF). For example, the Jun/BATF/IRF4 complex mediates chromatin accessibility in enhancers of genes that encode lineage-specific transcription factors and cytokine receptors in effector CD8^+^ T cells [1]. Jun is activated by phosphorylation by Jun N-terminal kinase (JNK) at Ser63 and Ser73 in Jun’s activation domain. This in turn results in the inhibition of both ubiquitination and degradation of Jun, allowing accumulation and increased transcriptional activity [2]. Jun then dimerises, either as a homodimer or as a heterodimer with other bZIP members, which binds to the 5′-TGAC/G TCA-3′ DNA sequence (known as the TPA response element, TRE) to promote transcription [3]. Jun is phosphorylated by other kinases such as glycogen synthase kinase (GSK) 3, which modifies Jun at Thr239, Ser342 and Ser249, and casein kinase (CK) 2, which phosphorylates Jun at Thr231 and Ser249 [4]. These sites are located proximally to the DNA-binding domain and are phosphorylated to inhibit the activity of Jun [4]. Jun levels are also indirectly regulated by other MAP kinases such as ERK, which was found to prevent Jun degradation by the inactivation of GSK3 [5]. ERK also controls Jun expression through its downstream activation of the cAMP response element (CRE) binding protein (CREB), which could potentially affect DNA binding elements essential for Jun transcription [5]. Developing evidence supports a duplicitous role for Jun, with capacity to either promote cancer growth or enhance anti-cancer immunity. Here, we review recent evidence challenging the traditional view of Jun as a pro-tumorigenic transcription factor.

## 2. Is Jun a Foe in Cancer?

Apoptotic evasion is a hallmark of cancer [6,7]. Berthenet et al. demonstrated that the JNK-AP1 transcriptional signalling axis was upregulated in WM852 metastatic melanoma cells surviving apoptosis, which resulted in increased melanoma migration, invasion and aggressiveness [8]. SP600125 (a potent and selective JNK inhibitor) and JNK1/2 siRNA reduced melanoma cell migration [8]. siRNA targeting Jun effectively reduced the growth of neuroblastoma cells [9]. In line with this, T5224, a compound that inhibits DNA binding of Fos and Jun, decreased glioblastoma cell viability, number, and clonogenicity [10]. This highlights the role of the AP-1 transcriptional family in promoting cancer aggressiveness. Han et al. found that Jun expression was associated with bone metastasis in luminal breast cancer [11] (Supplementary Tables S1 and S2). Jun deficiency in luminal breast cancer cells transplanted into mice resulted in decreased tumorigenesis and bone metastasis [11]. This led these investigators to surmise that inhibiting Jun, perhaps using a JNK inhibitor, may serve as an effective treatment strategy for luminal breast cancer [11]. Indeed, Jun has long been implicated as a therapeutic target for the treatment of a range of tumours using DNAzyme 13 (Dz13), a deoxyribozyme that cleaves *jun* mRNA [12]. A range of investigators, including ourselves, targeted *jun* using this approach in a range of animal models with efficacy against basal cell carcinoma, squamous cell carcinoma, melanoma, osteosarcoma, liposarcoma, breast cancer and prostate cancer [13,14,15,16]. For example, we found that Jun is expressed in >80% of primary and metastatic human melanoma cells [17]. Clinically, Dz13 decreased Jun expression in nine out of nine basal cell carcinomas in patients and reduced histological tumour depth in five out of nine [16] (Supplementary Table S3).

A key pathway involved in cancer pathogenesis is RAS/RAF/MEK/ERK (MAPK), a signalling pathway dysregulated in around 40% of all cancers [18]. Similarly, the PI3K pathway is also dysregulated in many human cancers [19]. Studies by Kappelmann-Fenzl et al. revealed that Jun promotes a malignant phenotype in melanoma cells by deregulating genes involved in the PI3K/AKT signalling pathway, such as *Bcl2*, *Ccnd1*, *Pdgfb*, *Cdk6*, and *Egfr;* the PI3K/AKT signalling pathway is crucial to cancer progression [20]. Interestingly, ERK signalling upregulates JNK which activates Jun, suggesting a close interplay between the two pathways which play an integral role in melanoma development [5].

Therapy based on inhibition of MAPK signalling is a standard treatment option for advanced melanoma [21]. However, RAF inhibitors (such as vemurafenib and dabrafenib) and MEK inhibitors (such as trametinib) are successful in the initial therapeutic phase but struggle to produce durable clinical benefit due to the development of resistance [22]. Interestingly, Ramsdale et al. report that Jun plays an important role in the development of MAPK treatment resistance in melanoma cells [23]. Crucially, they found that treating melanoma cells with a BRAF inhibitor induces Jun, which coincides with an epithelial–mesenchymal transition [23]. Similarly, overexpressing Jun in melanoma cells also induced epithelial–mesenchymal transition [23]. Importantly, combining a JNK inhibitor with a BRAF inhibitor was able to overcome MAPK inhibitor resistance, as demonstrated by decreased cell migration and increased cell death compared to melanoma cells when treated with a BRAF inhibitor alone [23]. The ability of Jun to enable melanoma cells to resist MAPK inhibitor treatment was also observed by Delmas et al. [24]. Additionally, studies have demonstrated that Jun is crucial for promoting tumour metastasis, specifically by promoting calcium-induced migration to bone, increased angiogenesis, directing the tumour microenvironment to promote migration and invasion, and increased tumour adhesion through fibrosis [11,25,26,27,28].

De-differentiated liposarcoma (DDLPS) is also associated with Jun amplification, with approximately 90% of DDLPS cases expressing Jun [29]. Sioletic et al. demonstrated that in vitro DDLPS cell migration and invasion is dependent on high levels of Jun. Additionally, Jun overexpression substantially increased in vivo growth of weakly tumorigenic DDLPS cell lines [30]. The investigators also concluded that although low levels of Jun may be sufficient for in vitro proliferation, high levels of Jun enhance DDLPS tumour invasiveness and growth in vivo [30].

Epidermal growth factor receptor (EGFR) is overexpressed in colorectal cancer and associated with aggressive tumour biology and poor prognosis. It also represents a therapeutic target, with monoclonal antibodies (e.g., cetuximab and panitumumab) demonstrating effective inhibition of this cell signalling pathway in wild-type RAS mutation positive patients, prolonging survival by 8.2 months [31]. Miao et al. demonstrated that overexpression of Jun in HCT116 colorectal cancer cells increased EGFR at both the mRNA and protein level, whilst analysis of tumour samples obtained from colorectal cancer patients identified a positive correlation between EGFR and Jun expression [32]. This may support the use of Jun as a negative prognostic biomarker in colorectal cancer. Notably, this is already the case in nasopharyngeal carcinoma (NPC), where Jun expression is significantly higher in NPC tissues when compared to normal nasopharyngeal mucosa tissue [33]. Furthermore, in vivo Jun knockdown suppressed NPC growth in xenograft mouse models [33].

Ferroptosis is a recently discovered type of cell death associated with iron accumulation and lipid peroxidation [34]. *O*-linked *N*-acetylglucosaminylation (*O*-GlcNAcylation) is a reversible post-translational modification which has been shown to play a significant role in cancer development [35]. Chen et al. reported that overexpression of *O*-GlcNAcylated Jun inhibits ferroptosis in liver cancer cells by stimulating glutathione synthesis; a positive association was also observed between *O*-GlcNAcylated Jun and glutathione synthesis in clinical liver cancer samples [36].

Yu et al. report that glycine amidinotransferase (GATM) is downregulated in cholangiocarcinoma (CCA) and low expression of GATM correlates with a poor prognosis [37]. Furthermore, GATM upregulation inhibited CCA cell proliferation in vitro and GATM overexpression inhibited CCA tumour growth in vivo [37]. Interestingly, they found an inverse relationship between GATM and the phosphorylation of JNK/Jun where GATM knockdown increased JNK/Jun phosphorylation and GATM overexpression decreased JNK/Jun phosphorylation [37]. Using the JNK activator anisomycin in GATM-overexpressing cells reversed the inhibitory effects on CCA growth by GATM overexpression, leading the authors to conclude that GATM overexpression inhibits CCA proliferation and aggressiveness by inhibiting JNK/Jun signalling [37].

Mitochondrial glutaminase (GLS) is a key driver for the metabolism of glutamine by catalysing the conversion of glutamine to glutamate in the tricarboxylic acid (TCA) cycle. An elevated GLS activity can therefore provide nutritional benefits for rapidly reproducing cells, such as cancer [38]. Lukey et al. demonstrated that Jun directly binds to the *GLS* promoter site and increases GLS activity in human breast cancer cells. The study also found that the overexpression of Jun significantly enhanced GLS expression, and sensitised cancer cells to GLS inhibitors [38].

### 2.1. Pro-Tumorigenic Role of Jun in the Tumor Microenvironment

Further complicating the role of Jun in cancer is the range of effects that it has on the tumour microenvironment (TME). Tumours are treated as a functional organ consisting of a range of cell types and molecules that contribute to the survival, growth, and spread of the cancer [39]. In addition to tumour cells, the TME consists of endothelial cells and their precursors, fibroblasts, myofibroblasts, immune cells, and smooth-muscle cells [40]. Targeting the TME is crucial in improving patient response to therapy and in preventing tumour growth and spread [41,42].

Due to its classification as a classical oncoprotein, research into the roles of Jun in cancer has predominately focused on its effects on tumour cells. However, this approach only partially considers the role the TME milieu plays upon tumorigenesis, necessitating a holistic appraisal of Jun’s role within this cellular milieu and its subsequent effects on tumour development and progression. This information will help us better understand the role Jun plays in malignancy.

### 2.2. Cancer-Associated Fibroblasts (CAFs)

TME fibroblasts mediate tumour initiation, progression, and metastasis [43]. Cancer-associated fibroblasts (CAFs) express and secrete signalling proteins which stimulate cancer cell proliferation, including insulin-like growth factor-1 (IGF-1), hepatocyte growth factor (HGF), stromal-cell-derived factor-1 (CXCL12), and a range of fibroblast growth factors (FGFs) [44]. CAFs have been shown to provide metabolic support for tumour cell survival and proliferation through the release of alanine, glutamine, deoxycytidine, proline, and lipid species [43,45,46]. Therefore, the interplay between CAFs and tumour cells plays a key role in cancer development and progression. Li et al. performed paired transcriptomic and epigenomic analysis of normal fibroblasts and CAFs derived from metastatic breast cancer patients and found that both *jun* and *fos* levels were enriched within the CAF population [47]. Furthermore, Jun protein was found to be highly enriched within the stroma of metastatic breast cancer. Of note, in vivo CAF exposure to a JNK inhibitor resulted in a non-inflammatory CAF profile, reduced stromal Jun expression and inhibition of metastasis [47]. These findings support the important, but detrimental role Jun plays in promoting CAF-mediated tumour metastasis in breast cancer. Additionally, Zhang et al. demonstrated that CAFs promote metastasis in hepatocellular carcinoma (HCC) via the secretion of fibronectin-1, a Jun-dependent pathway [25]. Therefore, evidence supports that Jun overexpression directs TME CAFs towards a pro-metastatic phenotype.

### 2.3. Tumor-Associated Macrophages (TAMs)

Macrophages are an abundant cell line within the TME, and in addition to their classical innate immune cell role, can polarize towards either an M1 (anti-tumorigenic) or M2 (pro-tumorigenic) phenotype within the TME, influencing tumorigenesis [48]. M2 phenotype TAMs have the capacity to suppress T cell activity, promoting tumour progression, angiogenesis, invasion, motility, and metastasis [49]. Currently, whether Jun plays an integral role in TAM phenotype differentiation is yet to be established. However, there is recent evidence supporting its pro-metastatic role in this cell type. Specifically, Cai et al. reported that Jun can bind to the promoter of allograft inflammatory factor 1 (A1F1) in M2-polarised macrophages, promoting A1F1 overexpression and proliferation of hepatoma cells in both in vitro and in vivo experiments [50]. Additionally, multivariate analysis of HCC tumours identified that A1F1 can serve as an independent prognostic marker of both disease-free and overall survival [50]. Notably, like CAFs, HCC TAMs have capacity to secrete fibronectin-1 through a Jun-dependent pathway, leading to increased metastasis, raising the question of whether this phenomenon may be conserved across all malignancies [25].

### 2.4. Myeloid-Derived Suppressor Cells

Myeloid-derived suppressor cells (MDSCs) are a highly diverse and loosely defined group of myeloid cells with immunoregulatory activity [51,52]. These cells are referred to as MDSC due to their myeloid origin and their specific ability to suppress the immune system in chronic disease, including cancer [51]. This suggests that MDSCs play a key role in ‘immunological evasion’, a cardinal characteristic of tumour development and progression [7,53]. Song et al. reported that in pancreatic cancer, pancreatic adenocarcinoma up-regulated factor (PAUF) activates the MAPK signalling pathway phosphorylating MEK1/2, ERK and JNK which leads to the activation and translocation of Jun and an increased release of immunosuppressive factors at the mRNA level [54]. Treating these cells with the MEK inhibitor PD98059 led to a reduction in Jun phosphorylation after PAUF treatment and a down-regulation of immunosuppressive factors at the transcript level [54]. In line with these findings, Lee et al. reported that co-culture of MDSCs with mesenchymal stromal cells (MSC) induced the MAPK signalling pathway, resulting in robust phosphorylation of JNK and a highly induced expression of immunosuppressive cytokines [55]. JNK is responsible for the phosphorylation of Jun as well as other transcription factors such as c-Myc, Elk-1 and ATF2 [56]. Treating these cells with SP600125 resulted in a decreased expression of these immunosuppressive cytokines [55]. Taken together, these findings suggest that Jun plays a role in regulating and promoting the immunoregulatory activity of MDSCs.

### 2.5. Angiogenesis

Angiogenesis is key to tumour development and proliferation, supporting the elevated metabolic requirements of the growing malignancy [53]. The importance of angiogenesis is exemplified by the benefit of inhibitory therapeutic agents (i.e., bevacizumab) and their FDA approval in multiple malignant processes [57,58].

Vleugel et al. reported that Jun is associated with proliferation and angiogenesis in invasive breast cancer [59]. They found that activated nuclear Jun was positively correlated with vascular endothelial growth factor (VEGF) levels and microvessel density [59]. Similarly, Zhang et al. found that Dz13 inhibited tumour vascular density in a preclinical model of melanoma (B16F10 melanoma mouse model) [15]. This decrease in angiogenesis in the presence of Dz13 resulted in reduced tumour size [15], suggesting an integral role for Jun in tumour angiogenesis [60].

## 3. Jun: Favourable in Cancer?

In conflict with its traditional role as an oncogene in malignancy, an oncomine analysis of 13 tumour data sets from a variety of malignancy types by Podar et al. demonstrated that nine tumour types had significantly lower *jun* gene expression when compared to normal cellular counterparts, while three of the tumour types had upregulated *jun* gene expression [61]. Furthermore, Podar et al. demonstrated that Jun upregulation in multiple myeloma inhibited proliferation and induced apoptosis, and that reduced Jun expression demonstrated tendency towards both a reduced overall survival and event-free survival in 11 of 67 multiple myeloma patients [61]. This highlights a duplicitous role for Jun in cancer. Indeed, other studies have demonstrated that Jun plays a role in the induction of apoptosis. Increased Jun activity is sufficient to trigger apoptotic cell death in NIH 3T3 fibroblasts [62]. Certainly, the ability for Jun to instigate cancer cell apoptosis implicates it as favourable in the cancer context.

Supporting these findings, a range of studies have demonstrated the link between increased JNK activation and the induction of apoptosis. Mhaidat et al. found that treating melanoma cells with the chemotherapeutic docetaxel led to the induction of apoptosis. Levels of apoptosis showed close correlation with levels of activated JNK in the cells [63]. Activation of JNK was paralleled by Jun activation [63]. That SP600125 was necessary for docetaxel’s ability to activate caspase 2 demonstrated JNK’s crucial role in inducing caspase 2-dependent apoptosis [63]. Similarly, Shieh et al. observed that doxycycline-induced apoptosis in melanoma cells was dependent on the activation of JNK [64]. Additionally, naringenin, caffeic acid phenethyl ester, hellebrigenin, plumbagin and theaflavin all induced apoptosis in a range of melanoma cells through the JNK pathway [65,66,67,68,69]. This supports Jun’s favourable role in cancer. Indeed, increased Jun activity was observed in melanoma cells undergoing apoptosis after treatment with plumbagin and theaflavin [68,69]. Entinostat is a histone deacetylase inhibitor that is currently in Phase I and Phase II clinical trials as a promising emerging treatment option for patients with advanced breast cancer [70]. Tanioka et al. found that *jun* knockdown in luminal breast cancer cell lines led to resistance to entinostat treatment [71]. Furthermore, *jun* knockdown led to an increase in Myc signalling; an oncogene involved in cell growth and proliferation which may be playing a major role in entinostat resistance [71,72]. Interestingly, *jun* copy number loss has clinical relevance for luminal breast cancer, as patients with *jun* copy number loss have worse prognoses and respond more poorly to hormonal therapies compared to patients with no *jun* copy number loss [71]. This suggests that Jun may have protective effects in luminal breast cancer, contrary to its traditional role as an oncogene. Interestingly, *jun* deletion in an in vivo model of K-Ras^G12D^-induced lung adenocarcinoma (LADC) was found to result in increased levels of JunD phosphorylation, which showed strong correlation with increased tumorigenesis [73]. In this study, Jun was tumour-suppressive, and its expression was inversely related to the JunD isoform in primary human LADC biopsy samples. The authors speculated that the JNK signalling pathway may exert both tumour-promoting and tumour-suppressive effects via JunD and Jun, respectively, and the varying outcomes of JNK signalling in different contexts may be due to balancing between both arms [73].

In the context of prostate cancer, elevated Jun levels are associated with higher survival probability in patients [74]. Jun was identified as a tumour suppressor by modulating the transcription of senescence and inflammation-associated genes [74]. Deletion of Jun in *Pten*-deficient mice results in reduced immune cell attraction, activation of Stat3 and IL-1β production, which accelerated tumour growth [74]. This suggests that Jun plays an important role in the regulation of anti-cancer immune responses.

## 4. New Opportunities in Immunotherapy

Recognition that the immune system plays an integral role in cancer control afforded medicine a paradigm shift in therapeutic strategies to manage cancer. These immunotherapies have improved disease-free and overall survival in a variety of malignancies, immune checkpoint inhibitors (ICIs) being the most effective. Humanised monoclonal antibody ICIs, including PD-1, CTLA-4, LAG-3 and TIM-3, target T cell immunoregulatory receptors utilised by tumours to suppress the immune response, promoting T cell mediated tumour recognition, cytotoxicity and death [75]. For example, ICIs have revolutionised the treatment of melanoma, increasing the 5-year overall survival rate of advanced disease from 5–10% to 30–40% [76,77]. To date, several ICIs have been developed and FDA-approved for a wide range of indications [78]. Though promising, the effectiveness of ICIs remains limited by response, resistance, toxicity and durability [79,80]. Notably, even the PD-1 ICI pembrolizumab has a response rate of approximately 33% in melanoma patients [81].

### 4.1. T Cell Activation

Environmental cues (i.e., oncogenes, growth factors and cytokines) can promote activation of the MAPK signalling pathway in T cells [82]. T cell receptor (TCR) activation ultimately results in JNK activation and an increase in AP-1 activity, promoting Jun/Fos heterodimer complexing with NFAT and binding to DNA to control the expression of key immune response molecules like interleukin-2 (IL-2) [82,83]. Activation of the CD28 co-stimulatory pathway augments the downstream effector cascades and enhances functions including IL-2 production and T cell survival by recruiting transcription factors such as AP-1 and NF-κB [82,84]. T cell activation also leads to subsequent expression of other co-stimulatory receptors such as 4-1BB (CD137), which mediates the interaction of T cells with antigen-presenting cells (APCs) by crosslinking of 4-1BB and 4-1BB ligand (4-1BBL) and promotes IL-2 production, differentiation and proliferation, as well as protecting against activation-induced cell death (ACID) of T cells [85]. Site-directed mutagenesis of AP-1 binding sites revealed the critical role of AP-1 in the regulation of 4-1BB expression in activated T cells [86].

Recently, Yukawa et al. demonstrated the relationship between AP-1 and chromatin accessibility during the early stages of T cell activation [87]. The study developed a dominant-negative inhibitor of AP-1 (named A-Fos) to prevent the formation and binding of the Fos/Jun complex, leading to decreased chromatin opening and remodelling at multiple AP-1 binding sites. Remarkably, more than 70% of the regions specifically accessible in activated T cells bound to AP-1 components such as JunB [87]. These collectively suggest the importance of AP-1 involvement in both the initiation and promotion of T cell-mediated immune responses.

### 4.2. T Cell Exhaustion

Interestingly, Fos and Jun are transiently activated in the TCR and co-stimulatory pathways whilst NFAT resides in the nucleus for extended periods. Reduced AP-1 levels during prolonged antigen exposure in the absence of co-stimulation can therefore drive an alternative transcription program, where “partnerless NFAT” continues to bind with target genes with lower transactivation potential. This shift is evidenced by the upregulation of inhibitory receptors including PD-1, CTLA-4, LAG-3 and TIM-3, which results in T cell exhaustion [82,85] and failure of their anti-tumour effector function [88]. This suggests that the stability and balance of the NFAT/AP-1 interaction is crucial within the process of T cell activation.

Studies aiming to identify genes induced by T cell exhaustion (by chronic infections) and PD-1 ligation uncovered BATF, which lies downstream of PD-1 and inhibits AP-1-mediated transcription by competitively dimerising with Jun (preventing the formation of a Fos/Jun heterodimer) [89]. Liu et al. recently discovered neuropilin-1 (Nrp-1) as another immune checkpoint marker which limits CD8^+^ T cell-mediated anti-tumour response, lowers persistence and promotes terminal exhaustion in tumour-infiltrated T cells [90]. Investigation on the role of NRP-1 in anti-cancer immunotherapy found that NRP-1 restricts the renewal of exhausted T cells by downregulating Jun activation upon TCR restimulation [90].

CD8^+^ memory T cells, derived from CD8^+^ effector T cells, will persist after an episode of antigen-specific stimulation and clonal expansion, affording an expedited and specific response to future antigen-specific exposure [91]. Comparatively, exhausted CD8^+^ T cells downregulate AP-1 proteins, including both Fos and JunB [91]. In genome-wide analysis, Jun was identified as one of 21 key transcription factors regulating genes coding for Memory T cell phenotype and function [92]. Furthermore, sampling of peripheral blood lymphocytes from HCC patients, previously inoculated with T cell-activating tumour-associated antigen-derived peptides, identified that long-lasting CD8^+^ T cells develop an effector memory phenotype characterised by high expression of *IL7r*, *Sell* and upregulation of AP-1 transcription factors including *Jun* and *JunB* [93]. Collectively, these findings support an essential role for Jun in both activating T cells and mitigating their exhaustion.

### 4.3. Jun Overexpression in T Cells

Chimeric antigen receptor (CAR) T cells have formed another pillar of immunotherapy, displaying effective and durable clinical responses [94]. CARs are synthetically designed and redirect lymphocytes to “*recognise*” and “*eliminate*” cells expressing the receptor specific target antigen [95]. Unfortunately, similar to ICI treatment, the efficacy of CAR T cell therapy is limited by T cell exhaustion and resultant paucity of clonal expansion and persistence [95].

Lynn et al. found that overexpression of *jun* in CAR T cells (using a retroviral vector) can overcome T cell exhaustion [96]. In five different in vivo tumour models, these CAR T cells demonstrated enhanced clonal expansion potential, increased functional capacity, reduced terminal differentiation and greater anti-tumour potency. Simultaneously, there was a decrease in markers of T cell exhaustion, namely PD-1 and CD39. These findings suggest that *jun* overexpression may abrogate the poor clonal expansion and persistence associated with exhaustion in CAR T cell therapy. These findings were corroborated by Hussein et al., whose TCR T cell study confirmed that overexpressing *jun* augmented activated CD8^+^ T cell expansion, improved their tumour infiltration, and increased their persistence in a murine model of HCC [97]. Additionally, these TCR T cells had reduced expression of the exhaustion markers LAG-3 and TIM-3 following tumour antigen stimulation [97]. An additional study by Heitzemeder et al. with CAR T cells demonstrated again that overexpression of *jun* results in significant improvement in potency and persistence, in this case against neuroblastoma, without apparent off-target toxicity [98].

Comparatively, Xu et al. found no significant effect on clonal expansion and anti-tumour activity in their ovarian cancer CAR T cell model exploring overexpression of Jun [99]. These authors concluded their results differed from those produced by Lynn et al., Hussein et al. and Heitzemeder et al., secondary to a lack of homology between the motifs of these CAR T cells. Interestingly, although a difference in persistence and proliferation was not observed in their study, *jun* overexpression promoted the central memory phenotype, slightly increased IL-2 production and reduced LAG-3 expression, suggesting favourable anti-tumour effects [99].

These preclinical findings have driven a Phase I acute lymphoid leukemia clinical trial investigating overexpression of *jun* in CAR T cell therapy [100]. Zuo et al. reported that CAR T cells have limited effector function in acute myeloid leukemia (AML) which can be rescued by *jun* overexpression in vivo [100]. *Jun*-overexpressing CAR T cells have demonstrated enhanced anti-tumour cytotoxicity, with increased clonal expansion rate, in an in vivo leukemia model [100]. In line with this, *jun*-overexpressing CAR T cells exhibited enhanced tumour lysis and increased IL-2 and IFN-γ production subsequent to in vitro co-culture with leukemia cells [100]. The authors then conducted a Phase I trial of *jun*-overexpressing CAR T cells to evaluate their safety and efficacy. The trial has been closed early secondary to safety concerns around cytokine release syndrome, dose-limiting toxicity and severe infection [100]. Notably, the investigators did confirm that their CAR T cells expanded significantly in all patients, also demonstrating preliminary activity within a clinical setting [100]. Taken together, these findings suggest that *jun* overexpression in T cells may optimise limitations associated with current immunotherapies.

### 4.4. Roles of Jun in Other Immune Cells

In addition to its beneficial effect on T cells, Jun is essential for the development and activity of dendritic cells (DC). Specifically, Novoszel et al. demonstrated that both Jun and JunB are required for CD8α cDC1 cell differentiation, a subset of conventional DC (cDC) [101]. Deletion of Jun and JunB therefore results in a loss of cDC1-dependent responses, including antigen cross-presentation for the activation of T cells [101]. Importantly, cDC1 cells are critical for anti-cancer immunity, increased CD8^+^ T cell tumour infiltration and improved patient survival in cancer [102].

Another cell type important in anti-cancer immunity is natural killer (NK) cells. scRNA analysis of human NK cells revealed three primary NK subsets: NK1, NK2 and NK3. The NK1 cluster was characterised with high levels of cytotoxic molecules such as granzyme B and perforin, as well as high expression of *jun* and *junB* [103]. Mgrditchian et al. discovered that the inhibition of autophagy can significantly enhance NK cell tumour infiltration [104]. Mechanistically, this study demonstrated that the specific silencing of the autophagy gene Beclin 1 (*Becn1*) resulted in increased phosphorylation of both JNK and Jun, where Jun then transcriptionally enhances the expression of Ccl5 (also known as RANTES), a chemokine promoting NK cell infiltration [104]. The study also identified that elevated Ccl5 expression, in patients suffering from melanoma, conferred a survival advantage [104].

### 4.5. Jun—A Double-Edged Sword in Immunotherapy?

Notwithstanding evidence supporting an anti-tumour immunity role, research has also demonstrated that Jun overexpression can drive tumour differentiation and proliferation. Yu et al. recently showed that Jun overexpression can increase PD-L1 mRNA and protein levels, and utilising ChIP confirmed the direct binding of Jun to the PD-L1 promoter [105]. PD-L1 is commonly expressed in cancer cells to interact with PD-1 on activated T cells and inhibit their activity, as a means of immune evasion [106]. Notably, siRNA knockdown of Jun resulted in a significant decrease of PD-L1 in melanoma cell lines [82] and reduced Jun expression using a novel Jun inhibitor, ailanthone, suppressed melanoma progression and inhibition of regulatory T cell (Treg) infiltration [105]. Tregs are immunosuppressive T cells that maintain immune homeostasis [107]. Mechanistically, Jun was found to enhance the expression of key proteins involved in the post-transcriptional [108] and post-translational [109] modification of PD-L1. For example, knockdown or inhibition of JNK1 resulted in the impairment of Jun binding to the RNA N^6^-methyladenosine (m^6^A) methyltransferase-like (METTL) 3 promoter, which decreased the RNA m^6^A levels on the PD-L1 mRNA, lowering its stability and expression [108]. In the context of nasopharyngeal carcinoma (NPC), Jun is activated by TGF-β1 and binds to the promoter of the N-glycosyltransferase STT3A, which allows N-glycosylation of PD-L1, leading to enhanced immune evasion [109]. In the context of cancer, Tregs may limit the efficacy of immunotherapy and result in resistance to treatment. For example, PD-1 blockade was found to expand Treg population in human melanoma [110]. Notably, JunB is critical for the suppressive functions of Treg cells and regulates key effector molecules such as CTLA-4 [107], which may result in unfavourable inhibition of the anti-tumour response.

## 5. Concluding Remarks

**Figure 1:**
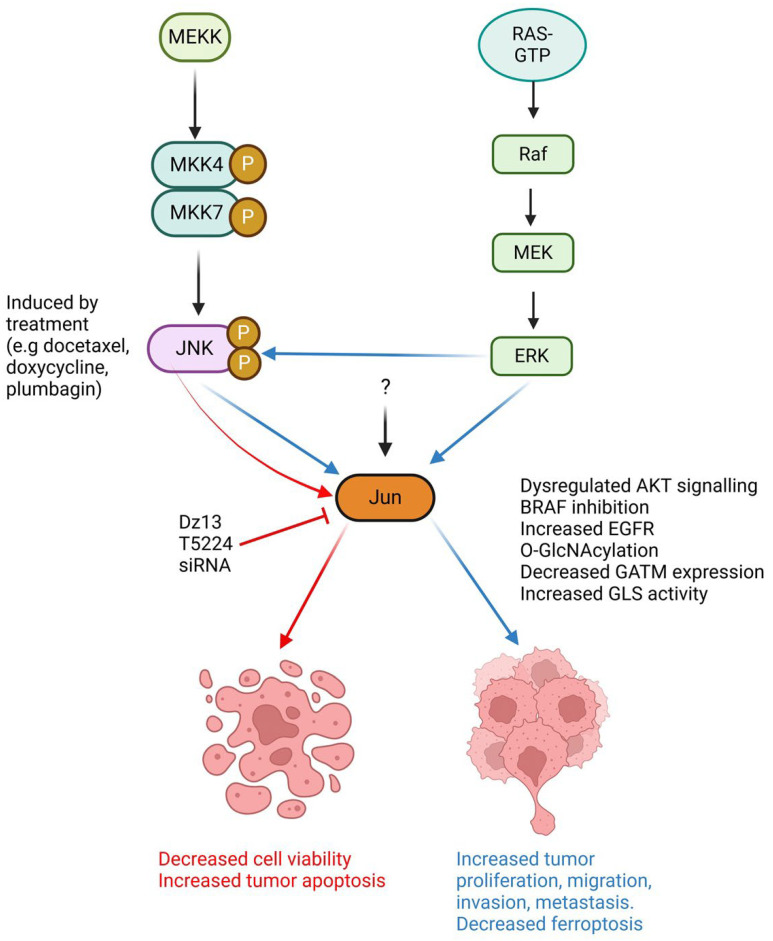
Simplified schematic demonstrating the duplicitous nature of Jun within cancer cells. JNK and ERK pathways activate Jun, although there may be other pathways that can also activate Jun (indicated by “?”). Jun expression can either promote tumour proliferation, migration, invasion, metastasis and inhibit ferroptosis (blue arrows) or alternatively, promote tumour apoptosis and reduce cell viability (red arrows). Figure were created using Biorender.com.

While Jun is a prototypic member of the ubiquitous AP-1 family of dimeric transcription factors regulating multiple cellular and physiological functions, mounting evidence suggests that Jun exhibits dual functionality in cancer, a regulatory protein with two faces (Figure 1). On one hand, Jun regulates the growth of multiple cancer types including skin cancers, breast cancer, nasopharyngeal carcinoma, liposarcoma, osteosarcoma and prostate cancer. Jun regulates tumour cell migration and invasiveness, as well as angiogenesis. It can also direct CAFs within the TME toward a pro-metastatic phenotype and control the immunoregulatory activity of MDSCs. Endogenous Jun elevation in tumour cells is linked to tumour progression, making it a potential therapeutic target. On the other hand, Jun can stimulate apoptosis in a range of cancer cell types and play a tumour-suppressing role, for example, in a manner inversely related to JunD or by transcriptional regulation of senescence- and inflammation-associated genes including IL-1β, TNFα, CCL3 and CCL8. In tumour cells, Jun elevation induced by treatment is often associated with tumour cell apoptosis and improved clinical outcomes. Overexpression of *jun* in CAR T cells and TCR T cells can prevent T cell exhaustion, and potentially abolish exhaustion-associated clonal expansion and persistence—challenges associated with CAR T cell therapy and other immunotherapies.

Whether Jun plays a favourable or unfavourable role in cancer is complex and context-dependent. For example, cancer cells with high basal Jun expression typically display a malignant phenotype. First, inhibition of Jun (e.g., Dz13, shRNA) or JNK (e.g., JNK-IN-8) is generally favourable as this suppresses cancer cell growth or invasion. Second, forced expression of Jun in tumour cells or the TME is typically unfavourable, as this appears to fuel the malignant phenotype, prevent cancer cell death, facilitate cancer cell growth and/or invasion. Third, when Jun expression is induced in cancer cells by compounds or agonists (e.g., adaphostin, naringenin, AIL), this is favourable as this can result in cancer cell apoptosis and suppressed cell growth. However, increased Jun expression in response to certain agonists (e.g., PAUF, CSF1) in TME cells can result in unfavourable effects including increased cancer cell migration or release of immunosuppressive cytokines. Lastly, forced expression of Jun in T cells is usually favourable, as this appears to reduce T cell exhaustion.

Future studies should delineate precise mechanisms underpinning Jun’s dual role in cancer, and how best to target Jun by way of under- or over-expression strategies, without causing adverse effects. The ultimate strategy will likely depend on the targeting approach, specific cancer type, genetic and phenotypic context, co-factors and possible involvement of collaborative and compensatory pathways.

## Supplementary Materials

The following supporting information can be downloaded at: https://www.mdpi.com/article/10.3390/ijms26020555/s1. References [111,112] are cited in the Supplementary Materials.
