# Supplementary material for: Jun, an Oncological Foe or Friend?
Source: Int J Mol Sci. 2025 Jan 10;26(2):555. doi: 10.3390/ijms26020555 (PMC11766113; doi:10.3390/ijms26020555)
Supplement: Supplementary file 1 [file ijms-26-00555-s001.zip › ijms-3369673-supplementary.pdf]

**SUPPLEMENTARY MATERIAL**

**Supplementary Table S1. *In vitro* studies exploring the role of Jun in cancer.** ND is not determined.

| ***In vitro* model** | **Cells used** | **Signalling pathway** | **Jun expression** | **Study outcome** | **Jun favorable or unfavorable in this cancer context** | **Limitations** | **Ref** |
|---|---|---|---|---|---|---|---|
| Tumor cells | MCF-7 luminal breast cancer cells, with a propensity for bone metastasis | JNK | High basal | Elevated Jun promotes malignant transformation and osteolytic bone metastasis of MCF7 cells | Unfavorable | Focused primarily on the interplay between breast cancer cells and osteoclasts, excluding the immune system and tumor microenvironment (TME) | Han *et al.* (2023) [1] |
| Tumor cells | 6 melanoma cell types (3 BRAF mutant, 3 PTEN mutant) | JNK, PI3K/AKT | High basal | Jun promotes a pro-survival and anti-apoptotic phenotype in melanoma cells via activation of the PI3K/AKT signalling pathway | Unfavorable | Study limited to tumor cells, thereby excluding the immune system and TME | Kappelmann-Fenzl *et al*. (2019) [2] |
| Tumor cells | 22 BRAFV600-mutant melanoma cell lines, including 4 cell lines intrinsically drug-resistant | JNK | High basal p-Jun (2 of 4), high basal total Jun levels (4 of 4) | Increased Jun promoted both BRAF inhibitor resistance and epithelial to mesenchymal cell transition | Unfavorable | Study limited to tumor cells, thereby excluding the immune system and TME | Ramsdale *et al*. (2015) [3] |
| Tumor cells | Dedifferentiated liposarcomas (DDLPS) - LP6 | ND | High basal | High Jun promotes proliferation, invasion and cell migration | Unfavorable | Study limited to tumor cells, thereby excluding the immune system and TME | Sioletic *et al.* (2014) [4] |
| Tumor cells | Liver cancer cell lines Bel-7402 and SMMC-7721 | ND | Forced overexpression | Increased Jun prevents ferroptosis of liver cancer cells | Unfavorable | Study limited to tumor cells, thereby excluding the immune system and TME | Chen *et al.* (2019) [5] |

| | | | | | | | |
|---|---|---|---|---|---|---|---|
| Tumor cells | Multiple myeloma (MM) cells | JNK | Adaphostin induced | Increased Jun in multiple myeloma cells inhibits proliferation and promotes apoptosis | Favorable | Study limited to tumor cells, thereby excluding the immune system and TME | Podar *et al*. (2007) [6] |
| Tumor cells | Human melanoma A2058 | JNK | Naringenin induced | Naringenin triggered cell apoptosis by phosphorylating JNK and Jun and stimulating cellular autophagy | Favorable | Study limited to tumor cells, thereby excluding the immune system and TME | Fernando *et al.* (2024) [7] |
| Tumor cells | Human breast cancer cells (luminal cell lines) | Myc | Knockdown or deletion | Jun knockdown increased Myc signalling and prevented entinostat inhibition of breast cancer | Favorable | Study limited to tumor cells, thereby excluding the immune system and TME; mouse melanoma | Tanioka *et al.* (2018) [8] |
| TME cells | Induced cancer associated fibroblasts (iCAFs) | JNK | Forced overexpression | Jun promotes a pro-metastatic fibroblast phenotype | Unfavorable | Study limited to CAFs, thereby excluding the immune system and TME | Li *et al.* (2022) [9] |
| TME cells | Tumor-associated macrophages (TAMs) | CSF1R, MEK1/2, ERK1/2, Jun | CSF1 induced | Jun binds the A1F1 promoter in macrophages which promotes hepatoma cell migration in a co-culture system | Unfavorable | Study limited to TAMs, thereby excluding the immune system and TME | Cai *et al.* (2017) [10] |
| TME cells | Myeloid-derived suppressor cells (MDSCs) | MEK, ERK1/2, JNK | Pancreatic adenocarcinoma up-regulated factor (PAUF) induced | Activation and translocation of Jun leading to increased release of immunosuppressive cytokines | Unfavorable | Study limited to MDSCs, thereby excluding the immune system and TME | Song *et al.* (2016) [11] |
| T cells | CAR T cells | ND | Forced overexpression | CAR T cells overexpressing *jun* had increased IFN-γ and IL-2 cytokine production | Favorable | Study limited to Jun overexpression in T cells only | Lynn *et al.* (2019) [12] |

| | | | | | | | |
|---|---|---|---|---|---|---|---|
| T cells | TCR T cells | ND | Forced overexpres sion | TCR T cells overexpressing *jun* had increased expansion potential in culture, higher tumor driven expansion with reduced exhaustion, and increased resistance to activation induced cell death | Favorable | Study limited to looking at Jun in T cells only | Hussein *et al*. (2023) [13] |
| T cells | CAR T cells | ND | Forced overexpres sion | CAR T cells overexpressing *jun* had increased killing against neuroblastoma cells *in vitro* | Favorable | Study limited to looking at Jun in T cells only | Heitzeneder *et al*. (2022) [14] |
| T cells | CAR T cells | ND | Forced overexpres sion | CAR T cells overexpressing *jun* had slightly increased IL-2 production and reduced LAG-3 expression | Favorable | Study limited to looking at Jun in T cells only | Xu *et al*. (2023) [15] |
| T cells | CAR T cells | ERK1/2 | Forced overexpres sion | CAR T cells overexpressing *jun* showed enhanced killing and increased IL-2 production when coincubated with U937 cancer cells. *jun* CAR T cells coincubated with U937 showed higher T cell activation, positive regulation of immune effector processes, and increased ERK1/2 signalling | Favorable | Study limited to looking at Jun in T cells only | Zuo *et al*. (2023) [16] |
| Tumor cells | Human and mouse melanoma cell lines | ND | Ailanthone (AIL) inhibition | AIL directly inhibited Jun leading to inhibition of melanoma growth, metastasis and invasion *in vitro*. AIL inhibited PD-L1 expression by inhibiting Jun | Unfavorable | Study limited to tumor cells | Yu *et al*. (2022) [17] |

**Supplementary Table S2.** ***In vivo*** **studies exploring the role of Jun in cancer.**

| ***In vivo* model** | **Tumor cells/type** | **Animal model** | **Animal model type** | **Immune status** | **Jun effect** | **Treatment** | **Study outcome** | **Jun favorable or unfavorable in this cancer context** | **Limitations** | **Ref** |
|---|---|---|---|---|---|---|---|---|---|---|
| Mice | Human luminal breast cancer, the highest bone metastasis | NOD/scid mice | Xenograft | Immunodeficient | Inhibition | JNK inhibitor, JNK-IN-8 | Jun inhibition using a JNK inhibitor effectively inhibits bone metastatic lesions of luminal breast cancer | Unfavorable | Immunodeficient model masks the effects of Jun in an immunocompetent system | Han *et al.* (2023) [1] |
| Mice | Human prostate cancer PC3, breast cancer MDA-MB231, osteosarcoma SaOS-2, G292 and osteosarcoma 143B cell lines | Balb/c nude mice | Xenograft | Immunodeficient | Inhibition | Dz13 | Dz13 inhibited tumor growth and metastasis | Unfavorable | Immunodeficient model masks the effects of Jun in an immunocompetent system | Tan *et al.* (2010) [18] |
| Mice | SW872 human iposarcoma cell line | Nude mice | Xenograft | Immunodeficient | Inhibition | Dz13 | Dz13 inhibited LS growth | Unfavorable | Immunodeficient model masks the effects of Jun in an immunocompetent system | Dass *et al.* (2008) [19] |
| Mice | B16F10 mouse melanoma cells | C57BL/J6 mice | Allograft | Immunocompetent | Inhibition | Dz13 | Dz13 inhibited solid tumor growth in | Unfavorable | Mouse allograft model | Zhang *et al.* (2004) [20] |

| | | | | | | | | | | |
|---|---|---|---|---|---|---|---|---|---|---|
| | | | | | | | mice injected with murine melanoma | | | |
| Mice | Mouse dermal T79 SCC (squamous cell carcinoma) tumor cells | SCID mice | Allograft | Immunodeficient | Inhibition | Dz13 | Dz13 inhibited T79 SCC growth in SCID mice | Unfavorable | Immunodeficient model masks the effects of Jun in an immunocompetent system | Cai *et al.* (2012) [21] |
| Mice | Human BCC-1/KMC cells (basal cell carcinoma cell line) | SCID mice | Xenograft | Immunodeficient | Inhibition | Dz13 | Dz13 inhibits BCC-1/KMC growth in SCID mice | Unfavorable | Immunodeficient model masks the effects of Jun in an immunocompetent system | Cai *et al.* (2012) [21] |
| Mice | Mouse UV13-1 SCC | C3H/HeN mice | Allograft | Immunocompetent | Inhibition | Dz13 | Dz13 inhibits SCC growth in immunocompetent mice involves cell-mediated immunity | Unfavorable | Mouse allograft model | Cai *et al.* (2012) [21] |
| Mice | Jun overexpressing human 141 and 510 liposarcomas cells | Nu/Nu mice | Xenograft | Immunodeficient | Overexpression | Overexpression | Jun overexpression enhances growth of liposarcoma *in vivo* | Unfavorable | Immunodeficient model masks the effects of Jun in an immunocompetent system | Sioletic *et al.* (2014) [4] |

| | | | | | | | | | | |
|---|---|---|---|---|---|---|---|---|---|---|
| Mice | Human radioresistant CNE-2R cells | Balb/c nude mice | Xenograft | Immunodeficient | Inhibition | shRNA | *jun* knockdown inhibited NPC progression *in vivo* | Unfavorable | Immunodeficient model masks the effects of Jun in an immunocompetent system | Sun *et al.* (2021) [22] |
| Mice | Mouse EO771-luc cells | C57BL/6 genetic background-wildtype or -*Jun*$^{fl/fl}$ mice | Allograft | Immunocompetent | Deficiency | *jun*$^{fl/fl}$ mice (*jun* deficiency) | *jun* deficiency in stroma inhibits cancer metastasis *in vivo* | Unfavorable | Mouse allograft model | Li *et al.* (2022) [9] |
| Mice | Human melanoma A375.S2 cells | Nude mice | Xenograft | Immunodeficient | Increased p-Jun (in vitro) | Plumbagin | Plumbagin inhibited growth of A375.S2 cells (increased apoptosis) in nude mice | Favorable | Immunodeficient model masks the effects of Jun in an immunocompetent system | Wang *et al.* (2008) [23] |
| Mice | Kelly neuroblastoma; EW8 Ewing's sarcoma; 143b and TC32 osteosarcoma | NSG mice | Xenograft | Immunocompetent | Forced expression | CAR T cells overexpressing *jun* | CAR T cells overexpressing *jun* showed increased functional capacity and improved anti-tumor potency, and improved expansion potential, and reduced expression | Favorable | Study did not explore role of Jun levels in tumor cells | Lynn *et al.* (2019) [12] |

| | | | | | | | | | | |
|---|---|---|---|---|---|---|---|---|---|---|
| | | | | | | | of T cell exhaustion markers in 5 different mouse models | | | |
| Mice | HepG2 hepatocellular carcinoma cells | NSG mice | Xenograft | Immunocompetent | Forced expression | TCR T cells overexpressing *jun* | TCR T cells overexpressing *jun* showed improved functional capacity against hepatocellular carcinoma and improved overall survival of the mice. These T cells displayed less exhaustion | Favorable | Study did not explore role of Jun levels in tumor cells | Hussein *et al*. (2023) [13] |
| Mice | Patein derived neuroblastoma cells | NSG mice | Xenograft | Immunocompetent | Forced expression | CAR T cells overexpressing *jun* | CAR T cells overexpressing *jun* displayed increased duration of disease control and CAR T cell persistence | Favorable | Study did not explore role of Jun levels in tumor cells | Heitzeneder *et al*. (2023) [14] |
| Mice | SKOV3 ovarian cancer cells | NSG mice | Xenograft | Immunocompetent | Forced expression | CAR T cells overexpressing *jun* | No improvement in CAR T cell anti-tumor activity but CAR T cells overexpressing *jun* had reduced LAG-3 expression | Favorable | Study did not explore role of Jun levels in tumor cells | Xu *et al*. (2022) [15] |
| Mice | U937 myeloid leukemia cells | NSG mice | Xenograft | Immunocompetent | Forced expression | CAR T cells | CAR T cells overexpressing *jun* had higher | Favorable | Study did not explore role of Jun levels | Zuo *et al*. (2024) [16] |

| | | | | | | | | | | |
|---|---|---|---|---|---|---|---|---|---|---|
| | | | | | | overexpressing *jun* | proliferation and improved anti-tumor activity | | in tumor cells | |
| Mice | B16F10 mouse melanoma cells | C57BL/6 mice | Allograft | Immunocompetent | Inhibition | AIL | AIL was screened a Jun inhibitor. AIL reduced tumor volume. AIL improved efficacy of anti-PD-L1 treatment | Unfavorable | Study focused on effects of Jun in tumor cells only | Yu *et al*. (2022) [17] |

**Supplementary Table S3. Clinical research exploring the role of Jun in cancer.** N/A denotes information not available.

| Data source | Tumor type | Patient number | Jun effect | Treat ment | Relations between Jun level and patient survival | Study outcome | Jun favorable or unfavorable in this cancer context | Limitations | Ref |
|---|---|---|---|---|---|---|---|---|---|
| Phase I Clinical trail | Nodular basal cell carcinoma (BCC) | 9 | Inhibition | Dz13 | N/A | Patients were treated with Dz13 which cleaves *jun* mRNA. Jun expression was reduced in 9 of 9 patients. 5 of the 9 patients had reduced tumor depth | Unfavorable | Non-controlled, non-randomized, non-blinded, dose-escalating Phase I clinical trial | Cho *et al.* (2013) [24] |
| Patient tissues | Colorectal cancer (CRC) | 6 | N/A | N/A | N/A | Jun expression was positively correlated with EGFR and RHBDD1 expression | Unfavorable | Analyzed by Western blotting, indirect effects of Jun on colorectal cancer | Miao *et al.* (2017) [25] |
| GEO and TCGA databases | Hepatocellular carcinoma | N/A | N/A | N/A | Survival analysis showed Jun expression increased, and HCC prognosis was worse | Jun and its network regulate HCC, mainly positively correlated with TAMs and fibroblasts | Unfavorable | Unclear whether the patients in this study received therapies that could affect Jun expression. Additionally, study focused on TAMs and fibroblasts, rather than tumor cells themselves | Zhang *et al.* (2022) [26] |
| Patient tissues | Liver cancer | 8 | N/A | N/A | N/A | Positive correlation between Jun O-GlcNAcylation and GSH was observed in clinical samples | Unfavorable | Indirect effects of Jun on liver cancer, unclear whether the increase in Jun was Endogenous or induced | Chen *et al.* (2019) [27] |

| | | | | | | | | | |
|---|---|---|---|---|---|---|---|---|---|
| Clinical genetic data | Multiple myeloma (MM) | 67 | Induced | Patients were treated with Total Therapy 2 (TT2) Protocol | Shorter event-free survival and overall survival in MM patients with low Jun levels compared with patients with normal or high Jun levels | Jun up-regulation causes caspase-mediated c-Abl cleavage, and cleaved 60 kDa c-Abl product inhibits MM growth and induces cell death | Favorable | Findings must be validated in larger studies | Carrasco *et al.* (2006) [28], Podar *et al.* (2007) [6] |
| Clinical biopsy tissue RNA-seq data | Lung adenocarcinoma and lung squamous cell carcinoma | 17 | Induced | PD-1 blockade therapy | N/A | Jun as a predictive response marker in PD-1 blockade therapy | Favorable | Findings should be validated in larger studies. No data for Jun and patients' survival | Wang *et al.* (2024) [29] |
| TCGA database | Lung cancer | 1925 | N/A | N/A | High Jun expression resulted in better prognosis | Jun expression predictive | Favorable | Unclear whether patients received therapies that could affect Jun expression | Wang *et al.* (2024) [29] |
| Human breast cancer datasets, TCGA and METABRIC | Primary breast tumor | 1091 | N/A | N/A | Patients with *jun* copy number loss had worse prognosis among luminal breast cancers in METABRIC | Jun copy number loss in human breast cancer patients prognostic of worse outcome | Favorable | Unclear whether patients received therapies that could affect Jun expression | Tanioka *et al.* (2018) [8] |
| Phase I | Acute myeloid leukemia | 4 | Forced expression | CAR T cells overexpressing *jun* | N/A | Trial halted due to safety concerns. CAR T cells overexpressing *jun* expanded significantly and | Inconclusive | Small sample size, dose limiting Phase 1 trial. Difficult to draw conclusions | Zuo *et al.* (2024) [16] |

| | | | | | | | | | |
|---|---|---|---|---|---|---|---|---|---|
| | | | | | | showed some preliminary activity in patients | | | |

## References


1. Han, Y.; Katayama, S.; Futakuchi, M.; Nakamichi, K.; Wakabayashi, Y.; Sakamoto, M.; Nakayama, J.; Semba, K., Targeting c-Jun Is a Potential Therapy for Luminal Breast Cancer Bone Metastasis. *Molecular Cancer Research* **2023,** 21, (9), 908-921.
2. Kappelmann-Fenzl, M.; Gebhard, C.; Matthies, A. O.; Kuphal, S.; Rehli, M.; Bosserhoff, A. K., C-Jun drives melanoma progression in PTEN wild type melanoma cells. *Cell Death & Disease* **2019,** 10, (8), 584.
3. Ramsdale, R.; Jorissen, R. N.; Li, F. Z.; Al-Obaidi, S.; Ward, T.; Sheppard, K. E.; Bukczynska, P. E.; Young, R. J.; Boyle, S. E.; Shackleton, M.; Bollag, G.; Long, G. V.; Tulchinsky, E.; Rizos, H.; Pearson, R. B.; McArthur, G. A.; Dhillon, A. S.; Ferrao, P. T., The transcription cofactor c-JUN mediates phenotype switching and BRAF inhibitor resistance in melanoma. *Science Signaling* **2015,** 8, (390), ra82-ra82.
4. Sioletic, S.; Czaplinski, J.; Hu, L.; Fletcher, J. A.; Fletcher, C. D.; Wagner, A. J.; Loda, M.; Demetri, G. D.; Sicinska, E. T.; Snyder, E. L., c-Jun promotes cell migration and drives expression of the motility factor ENPP2 in soft tissue sarcomas. *J Pathol* **2014,** 234, (2), 190-202.
5. Chen, Y.; Zhu, G.; Liu, Y.; Wu, Q.; Zhang, X.; Bian, Z.; Zhang, Y.; Pan, Q.; Sun, F., O-GlcNAcylated c-Jun antagonizes ferroptosis via inhibiting GSH synthesis in liver cancer. *Cell Signal* **2019,** 63, 109384.
6. Podar, K.; Raab, M. S.; Tonon, G.; Sattler, M.; Barilà, D.; Zhang, J.; Tai, Y.-T.; Yasui, H.; Raje, N.; DePinho, R. A.; Hideshima, T.; Chauhan, D.; Anderson, K. C., Up-Regulation of c-Jun Inhibits Proliferation and Induces Apoptosis via Caspase-Triggered c-Abl Cleavage in Human Multiple Myeloma. *Cancer Research* **2007,** 67, (4), 1680-1688.
7. Fernando, P.; Zhen, A. X.; Piao, M. J.; Herath, H.; Kang, K. A.; Yoon, S. P.; Boo, H. J.; Hyun, C. L.; Hyun, J. W., Naringenin Induces Cellular Apoptosis in Melanoma Cells via Intracellular ROS Generation. *Anticancer Res* **2024,** 44, (3), 1079-1086.
8. Tanioka, M.; Mott, K. R.; Hollern, D. P.; Fan, C.; Darr, D. B.; Perou, C. M., Identification of Jun loss promotes resistance to histone deacetylase inhibitor entinostat through Myc signaling in luminal breast cancer. *Genome Med* **2018,** 10, (1), 86.

9. Li, Q.; Lv, X.; Han, C.; Kong, Y.; Dai, Z.; Huo, D.; Li, T.; Li, D.; Li, W.; Wang, X.; Zhao, Q.; Ming, J.; Yang, W.; Chen, Y.; Wu, X., Enhancer reprogramming promotes the activation of cancer-associated fibroblasts and breast cancer metastasis. *Theranostics* **2022,** 12, (17), 7491-7508.
10. Cai, H.; Zhu, X.-D.; Ao, J.-Y.; Ye, B.-G.; Zhang, Y.-Y.; Chai, Z.-T.; Wang, C.-H.; Shi, W.-K.; Cao, M.-Q.; Li, X.-L.; Sun, H.-C., Colony-stimulating factor-1-induced AIF1 expression in tumor-associated macrophages enhances the progression of hepatocellular carcinoma. *OncoImmunology* **2017,** 6, (9), e1333213.
11. Song, J.; Lee, J.; Kim, J.; Jo, S.; Kim, Y. J.; Baek, J. E.; Kwon, E. S.; Lee, K. P.; Yang, S.; Kwon, K. S.; Kim, D. U.; Kang, T. H.; Park, Y. Y.; Chang, S.; Cho, H. J.; Kim, S. C.; Koh, S. S.; Kim, S., Pancreatic adenocarcinoma up-regulated factor (PAUF) enhances the accumulation and functional activity of myeloid-derived suppressor cells (MDSCs) in pancreatic cancer. *Oncotarget* **2016,** 7, (32), 51840-51853.
12. Lynn, R. C.; Weber, E. W.; Sotillo, E.; Gennert, D.; Xu, P.; Good, Z.; Anbunathan, H.; Lattin, J.; Jones, R.; Tieu, V.; Nagaraja, S.; Granja, J.; de Bourcy, C. F. A.; Majzner, R.; Satpathy, A. T.; Quake, S. R.; Monje, M.; Chang, H. Y.; Mackall, C. L., c-Jun overexpression in CAR T cells induces exhaustion resistance. *Nature* **2019,** 576, (7786), 293-300.
13. Hussein, M. S.; Li, Q.; Mao, R.; Peng, Y.; He, Y., TCR T cells overexpressing c-Jun have better functionality with improved tumor infiltration and persistence in hepatocellular carcinoma. *Front Immunol* **2023,** 14, 1114770.
14. Heitzeneder, S.; Bosse, K. R.; Zhu, Z.; Zhelev, D.; Majzner, R. G.; Radosevich, M. T.; Dhingra, S.; Sotillo, E.; Buongervino, S.; Pascual-Pasto, G.; Garrigan, E.; Xu, P.; Huang, J.; Salzer, B.; Delaidelli, A.; Raman, S.; Cui, H.; Martinez, B.; Bornheimer, S. J.; Sahaf, B.; Alag, A.; Fetahu, I. S.; Hasselblatt, M.; Parker, K. R.; Anbunathan, H.; Hwang, J.; Huang, M.; Sakamoto, K.; Lacayo, N. J.; Klysz, D. D.; Theruvath, J.; Vilches-Moure, J. G.; Satpathy, A. T.; Chang, H. Y.; Lehner, M.; Taschner-Mandl, S.; Julien, J.-P.; Sorensen, P. H.; Dimitrov, D. S.; Maris, J. M.; Mackall, C. L., GPC2-CAR T cells tuned for low antigen density mediate potent activity against neuroblastoma without toxicity. *Cancer Cell* **2022,** 40, (1), 53-69.e9.
15. Xu, T.; Wang, C.; Chen, X.; Bai, J.; Wang, E.; Sun, M., Coexpression of C-Jun in Multiple-Chain DAP-CAR-engineered T-Cells for Solid Tumor Therapy. *Immunotherapy* **2022,** 14, (18), 1457-1466.

16. Zuo, S.; Li, C.; Sun, X.; Deng, B.; Zhang, Y.; Han, Y.; Ling, Z.; Xu, J.; Duan, J.; Wang, Z.; Yu, X.; Zheng, Q.; Xu, X.; Zong, J.; Tian, Z.; Shan, L.; Tang, K.; Huang, H.; Song, Y.; Niu, Q.; Zhou, D.; Feng, S.; Han, Z.; Wang, G.; Wu, T.; Pan, J.; Feng, X., C-JUN overexpressing CAR-T cells in acute myeloid leukemia: preclinical characterization and phase I trial. *Nat Commun* **2024,** 15, (1), 6155.
17. Yu, P.; Wei, H.; Li, K.; Zhu, S.; Li, J.; Chen, C.; Zhang, D.; Li, Y.; Zhu, L.; Yi, X.; Liu, N.; Liu, P.; Zhao, S.; Chen, X.; Peng, C., The traditional chinese medicine monomer Ailanthone improves the therapeutic efficacy of anti-PD-L1 in melanoma cells by targeting c-Jun. *Journal of Experimental & Clinical Cancer Research* **2022,** 41, (1), 346.
18. Tan, M. L.; Choong, P. F.; Dass, C. R., Direct anti-metastatic efficacy by the DNA enzyme Dz13 and downregulated MMP-2, MMP-9 and MT1-MMP in tumours. *Cancer Cell Int* **2010,** 10, 9.
19. Dass, C. R.; Galloway, S. J.; Clark, J. C. M.; Khachigian, L. M.; Choong, P. F. M., Involvement of c-jun in human liposarcoma growth: supporting data from clinical immunohistochemistry and DNAzyme efficacy. *Cancer Biology & Therapy* **2008,** 7, (8), 1297-1301.
20. Zhang, G.; Dass, C. R.; Sumithran, E.; Di Girolamo, N.; Sun, L.-Q.; Khachigian, L. M., Effect of Deoxyribozymes Targeting c-Jun on Solid Tumor Growth and Angiogenesis in Rodents. *JNCI: Journal of the National Cancer Institute* **2004,** 96, (9), 683-696.
21. Cai, H.; Santiago, F. S.; Prado-Lourenco, L.; Wang, B.; Patrikakis, M.; Davenport, M. P.; Maghzal, G. J.; Stocker, R.; Parish, C. R.; Chong, B. H.; Lieschke, G. J.; Wong, T.-W.; Chesterman, C. N.; Francis, D. J.; Moloney, F. J.; Barnetson, R. S. C.; Halliday, G. M.; Khachigian, L. M., DNAzyme Targeting c-jun Suppresses Skin Cancer Growth. *Science Translational Medicine* **2012,** 4, (139), 139ra82-139ra82.
22. Sun, Y.; Chen, K.; Lin, G.; Wan, F.; Chen, L.; Zhu, X., Silencing c-Jun inhibits autophagy and abrogates radioresistance in nasopharyngeal carcinoma by activating the PI3K/AKT/mTOR pathway. *Ann Transl Med* **2021,** 9, (13), 1085.
23. Wang, C. C. C.; Chiang, Y.-M.; Sung, S.-C.; Hsu, Y.-L.; Chang, J.-K.; Kuo, P.-L., Plumbagin induces cell cycle arrest and apoptosis through reactive oxygen species/c-Jun N-terminal kinase pathways in human melanoma A375.S2 cells. *Cancer Letters* **2008,** 259, (1), 82-98.

24. Cho, E. A.; Moloney, F. J.; Cai, H.; Au-Yeung, A.; China, C.; Scolyer, R. A.; Yosufi, B.; Raftery, M. J.; Deng, J. Z.; Morton, S. W.; Hammond, P. T.; Arkenau, H. T.; Damian, D. L.; Francis, D. J.; Chesterman, C. N.; Barnetson, R. S.; Halliday, G. M.; Khachigian, L. M., Safety and tolerability of an intratumorally injected DNAzyme, Dz13, in patients with nodular basal-cell carcinoma: a phase 1 first-in-human trial (DISCOVER). *Lancet* **2013,** 381, (9880), 1835-43.
25. Miao, F.; Zhang, M.; Zhao, Y.; Li, X.; Yao, R.; Wu, F.; Huang, R.; Li, K.; Miao, S.; Ma, C.; Ju, H.; Song, W.; Wang, L., RHBDD1 upregulates EGFR via the AP-1 pathway in colorectal cancer. *Oncotarget* **2017,** 8, (15), 25251-25260.
26. Zhang, L.; Zhang, C.; Xing, Z.; Lou, C.; Fang, J.; Wang, Z.; Li, M.; He, H.; Bai, H., Fibronectin 1 derived from tumor-associated macrophages and fibroblasts promotes metastasis through the JUN pathway in hepatocellular carcinoma. *International Immunopharmacology* **2022,** 113, 109420.
27. Chen, Y.; Zhu, G.; Liu, Y.; Wu, Q.; Zhang, X.; Bian, Z.; Zhang, Y.; Pan, Q.; Sun, F., O-GlcNAcylated c-Jun antagonizes ferroptosis via inhibiting GSH synthesis in liver cancer. *Cellular Signalling* **2019,** 63, 109384.
28. Carrasco, D. R.; Tonon, G.; Huang, Y.; Zhang, Y.; Sinha, R.; Feng, B.; Stewart, J. P.; Zhan, F.; Khatry, D.; Protopopova, M.; Protopopov, A.; Sukhdeo, K.; Hanamura, I.; Stephens, O.; Barlogie, B.; Anderson, K. C.; Chin, L.; Shaughnessy, J. D., Jr.; Brennan, C.; Depinho, R. A., High-resolution genomic profiles define distinct clinico-pathogenetic subgroups of multiple myeloma patients. *Cancer Cell* **2006,** 9, (4), 313-25.
29. Wang, Y.; Ran, T.; Li, Y.; Tian, L.; Yang, L.; Liu, Z.; Yao, B., Identification of JUN gene and cellular microenvironment in response to PD-1 blockade treatment in lung cancer patients via single-cell RNA sequencing. *Aging (Albany NY)* **2024,** 16, (12), 10348-10365.
